# Five years of radiographic evaluation for the peri-implant bone changes of all-on-four implant prostheses constructed from different framework materials using different digital construction techniques

**DOI:** 10.1186/s12903-024-04642-7

**Published:** 2024-08-07

**Authors:** Khloud Ezzat Mourad, Noha Hassan Atwa Hassan Rashed, Gilan Youssef Altonbary, Salah Abdel Fattah Hegazy

**Affiliations:** Department of Prosthodontics, Faculty of Dentistry, Mansoura University, Mansoura, Egypt

**Keywords:** CAD/CAM, PEEK, Soft metal, Selective laser melting, All-on-4

## Abstract

**Background:**

There is insufficient evidence recommending a framework material and a CAD/CAM manufacturing technique for mandibular implant-supported prostheses. The study objective was to evaluate the clinical application of different materials and construction techniques used for mandibular All-on-4 prosthesis on circumferential peri-implant bony changes after 5 years.

**Methods:**

Thirty-six male patients with all-on-4 mandibular implant-supported prostheses were recalled and divided into three groups. Group PK (patients with frameworks milled from PEEK blocks), Group PSM (patients with frameworks milled from soft metal blocks), and Group SLM (patients with frameworks constructed with additive manufacturing; selective laser melting). The circumferential bone level on all implant faces was assessed with a CBCT. Two-way repeated measures ANOVA was used to compare vertical bone loss (VBL) and horizontal bone loss (HBL) between different groups, implant positions, and observation times followed by Tukey’s multiple comparisons.

**Results:**

For all observation times, there was a significant difference in VBL between groups for both anterior and posterior implants (*P* < .001). For anterior implants, group PSM showed the lowest VBL while group PK showed the highest for anterior and posterior implants. For all groups, HBL significantly increased after 5 years for both anterior and posterior implants (*P* < .001). For anterior implants, group PSM showed the highest HBL. For posterior implants, group PK and SLM showed the highest.

**Conclusion:**

Within the study’s limitations, mandibular implant-supported fixed frameworks fabricated with either milling from PEEK or soft metal blocks, or additive manufacturing (laser melting technology) exhibited significant vertical and horizontal bone height changes after 5 years.

**Clinical Trial Registry Number:**

(NCT06071689) (11/10/2023).

## Background

Patients who struggle with complete dentures may benefit from rehabilitation with dental implants supporting a fixed full-arch prosthesis [1]. The “All-on-four” concept has been suggested to utilize as much residual alveolar bone as possible, permitting a rapid function and evading regeneration techniques that raise treatment costs, and have inherent risks [2]. A proper treatment approach that takes into account both the surgical and prosthodontic parts of the rehabilitation is necessary for this treatment to be successful over the long term [3]. To ensure the success of the implant-supported prosthesis, it is essential to comprehend the biomechanical principles to prevent the overloading of the bone and subsequent implant failure [4]. Various factors can affect the stresses on an implant and subsequent bone loss, which are typically categorized as either systemic- or patient-related factors (such as the patient’s overall health, age, smoking habits, oral hygiene maintenance), site- and implant-related factors (such as the location of the implant, the quality, and quantity of bone, the surface characteristics of the implant, its height and diameter), prosthesis-related factors (such as prosthesis material and construction technique that would affect marginal accuracy and passive fit), and the experience of the operator performing the implant procedure [5, 6]. In this situation, the framework material, geometry, and perfect fit of the prosthesis affect how much stress is placed on the bone surrounding the implant [7, 8]. The prosthesis framework’s role is to splint the implants together for support, permitting a more favorable transfer of load on the implants [9].

The use of computer-aided design/computer-aided manufacture (CAD/CAM) technology in dentistry has improved the precision of implant-supported prostheses [10]. Both subtractive and additive manufacturing technologies are currently used in the manufacture of frameworks [11]. Titanium and hard Co-Cr alloy are often used for implant frameworks because they have excellent biocompatibility, corrosion resistance, and machining capabilities for CAD-CAM [12, 13]. The selection of prosthetic material can be a matter of debate. As per Skalak et al., [14] utilizing hard prosthetic material; for the framework and/or occlusal surface; can lead to high-intensity loading between the implant and the supporting bone. On the other hand, using a material with a low modulus of elasticity can absorb stress and prevent possible damage to the surrounding bone caused by the load’s magnitude [15].

Co-Cr alloy metal frameworks with good structural homogeneity can be produced by milling solid metal blanks [16]. However, hard metal milling demands longer manufacturing times and higher costs due to the quick wear of milling tools [17]. As a result, Pre-sintered Soft Metal milling (PSM), a milling technology alternative to hard milling, has been created by compressing metal powder under isostatic pressure [16]. It allowed for reduced production time and prevented the milling equipment from wearing out quickly [18, 19]. PSM needs an extra sintering step after milling to achieve full density [20, 21]. Selective laser melting (SLM) is an additive manufacturing process that uses a powerful laser beam to fuse tiny layers of metal powder to create metal components directly from a 3D CAD model [22].

Polyetheretherketone (PEEK) has become a viable alternative to metal in dental treatments [23–25]. Although the elastic modulus of metal frameworks falls between 100 and 200 GPa, PEEK has an approximate modulus of 4 GPa [26]. Despite the difference in mechanical properties, PEEK is considered a viable option for the prosthesis frameworks on implants [27–29]. Peri-implant bone loss is regarded as a reliable indication of implant success and bone response to implant loading [30]. This study investigates the clinical application of less rigid materials for the All-on-4 prosthesis framework. The null hypothesis assumed that there wouldn’t be any difference between the three materials concerning the values of bone change surrounding dental implants for manufactured prostheses.

## Methods

### Study design

This is a retrospective study, in which patients with maxillary and mandibular fully edentulous arches, or a partially edentulous arch in need of extraction of the remaining compromised teeth, were rehabilitated with the maxillary complete denture and the All-on-4 implant distribution in the mandibular arch with different framework materials of the definitive prostheses. The primary objective was to assess the change in marginal bone levels of implants placed in these patients through 5 years period. The ethical committee (approval no.M0103023RP) approved the study protocol and was registered at www.clinicaltrials.gov (NCT06071689)(07/10/2023).

### Data collection

Dental records of patients who were treated from September 1, 2018, to October 20, 2023, were screened for inclusion. The inclusion criteria for radiographic data collection were: (1) Healthy male patients and free from any systemic diseases that may affect bone health, such as uncontrolled diabetes mellitus and osteoporosis (2) Implants placed with the All-on-4 concept in the mandible (3) CBCT baseline radiograph (3 to 4 months after implant placement; time of delivery of definitive implant-supported restoration) (4) CBCT follow-up radiographs (annual up to 5 years after definitive prosthesis insertion) (5) Regular check-ups for prosthetic maintenance (occlusal adjustments, screw loosening) of the prosthesis and monitoring compliance to oral hygiene measures. All included patients were well-maintained and were recalled every six months for the first two years and then yearly. The search included implants placed from September 1, 2018, to October 20, 2023. Individual records were excluded if: (1) they only had panoramic radiographs, (2) they did not have follow-up radiographs, or (3) patients’ records with a history of parafunctional habits (bruxism and clenching), smoking, and alcoholism.

All patients who wore All-on-4 implant prostheses were recalled after five years of their prosthesis insertion. Patients recalled from the database set of the clinic of the prosthodontic department. The patients were grouped based on the material of the framework used. All selected patients were treated with the same oral surgeon for implant placement. Patients also had the following criteria: four mandibular implants (3.6*14 mm; Dentium Superline II, Dentium, Co.) were placed according to All-on-4 distribution (two anterior straight implants and two posterior distally inclined implants; by 30 degrees relative to the occlusal plane). All patients had an opposing complete edentulous maxillary arch and attended the previous follow-up recalls with previous CBCT examinations. Patients who didn’t attend previous follow-up recalls, didn’t perform radiographic follow-up, or had para-functional habits were excluded from the study. Thirty-six male patients were selected to avoid any possible gender difference in masticatory performance or occlusal forces. Patients we divided into three groups; Group PK included twelve patients with frameworks that were milled from the PEEK block, Group PSM included twelve patients with frameworks milled from the soft metal block and Group SLM included twelve patients with frameworks fabricated with additive manufacturing using selective laser melting.

**Fig. 1:**
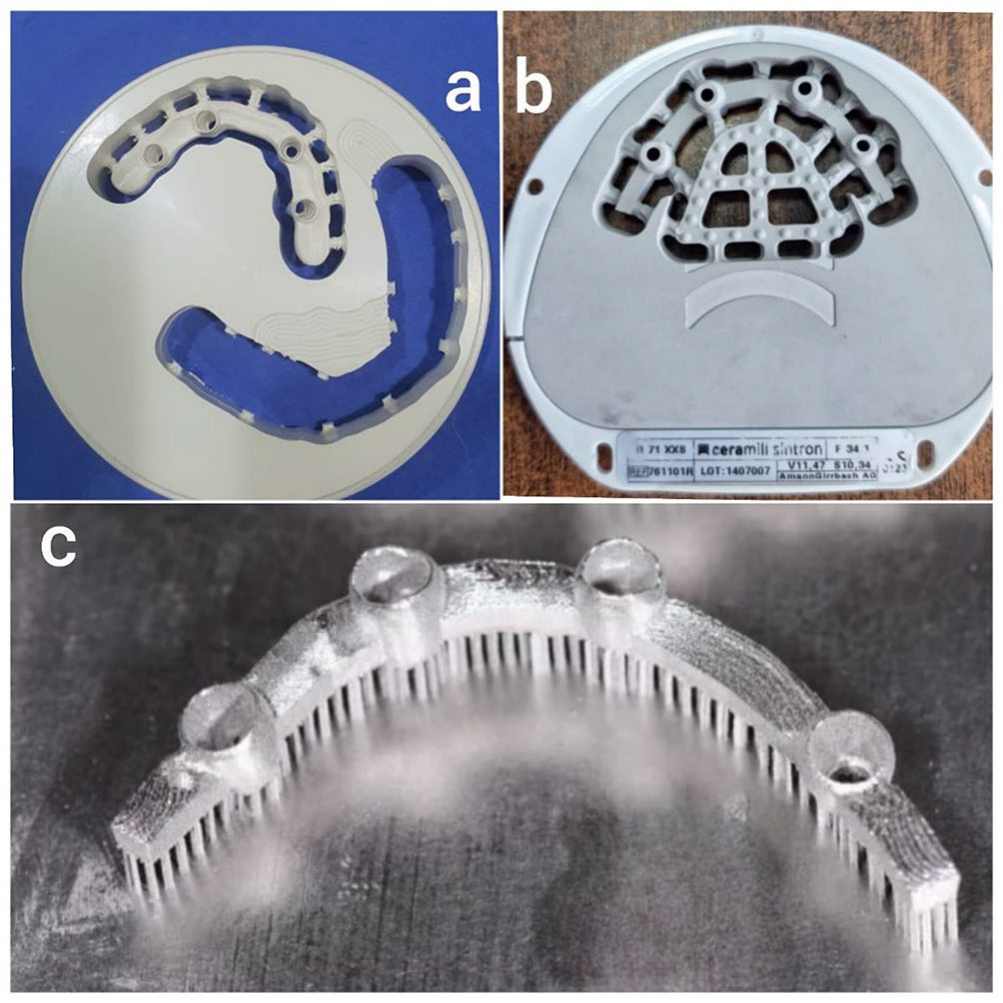
Construction of All-on-4 prosthesis frameworks. **a**: PEEK framework milled from PEEK block. **b**: Milled soft metal framework. **c**: 3-D printed selective laser melting of Co-Cr framework

The interventions (both surgical and prosthetic protocols for All-on-4 rehabilitation) have been shown in previous publications [25, 31]. In brief, the definitive prosthetic protocol was to construct a mandibular implant-supported prosthesis against a conventional acrylic maxillary complete denture. The prosthesis framework was manufactured according to the technique selected for each group. Group PK; The PEEK (BreCAM. BioHPP, Bredent GmbH & Co.; Modulus of elasticity 4.200–4.800 MPa, hardness 30 HV = 294 N/mm², Flexural strength 180–185 MPa) framework was milled using CAD-CAM machine following the CAD-CAM guidelines for design dimensions [32, 33]. (Fig. 1a) Group PSM; The framework was milled by dry Milling of CO-CR soft metal blocks (Ceramill Sintron, Modulus of elasticity 200 GPa, Vickers hardness 270 HV10, Tensile strength (Rm) 900 MPa) followed by sintering at 1280 °C for 5 h in a sintering oven under an argon atmosphere (Fig. 1b). For group SLM; the framework was constructed using the selective laser melting technique [31, 34]. CAD data of the design of the framework were forwarded to a laser melting machine (VULCAN TECH, Vm 120 PBF-LB AM machine) to construct the framework from the Co-Cr alloy powder (Starbond Easy Powder 30 g, Elastic modulus 225GPa, Vickers hardness 425 HV 10, Ultimate tensile strength1090MPa) with a 200 w Air Cooling Fiber Laser. 3D printing of the framework was done as the Co-Cr powder was applied to a stainless-steel plate and laser melted upward in subsequent layers, each of 20 mm in thickness, until the definitive product was generated [31, 34] (Fig. 1c).

All frameworks were designed to have a convex (rounded/teardrop-shaped) fitting surface; so that brushing and flossing can successfully remove the plaque and food debris [35, 36]. The framework dimensions were with a minimum anterior buccal-lingual width of 4 mm, a minimum occlusal cervical height of 5 mm, an increased width in the areas of the titanium sleeve to allow 6 mm of minimum buccal-lingual width and a minimum of 1–2 mm of acrylic resin with considering crown/implant ratio not exceeding 1.5-2 as recommended by Bayraktar et al. [37]. Prosthesis frameworks included 12 teeth, One-unit cantilever (< 10 mm) [38, 39].

**Fig. 2:**
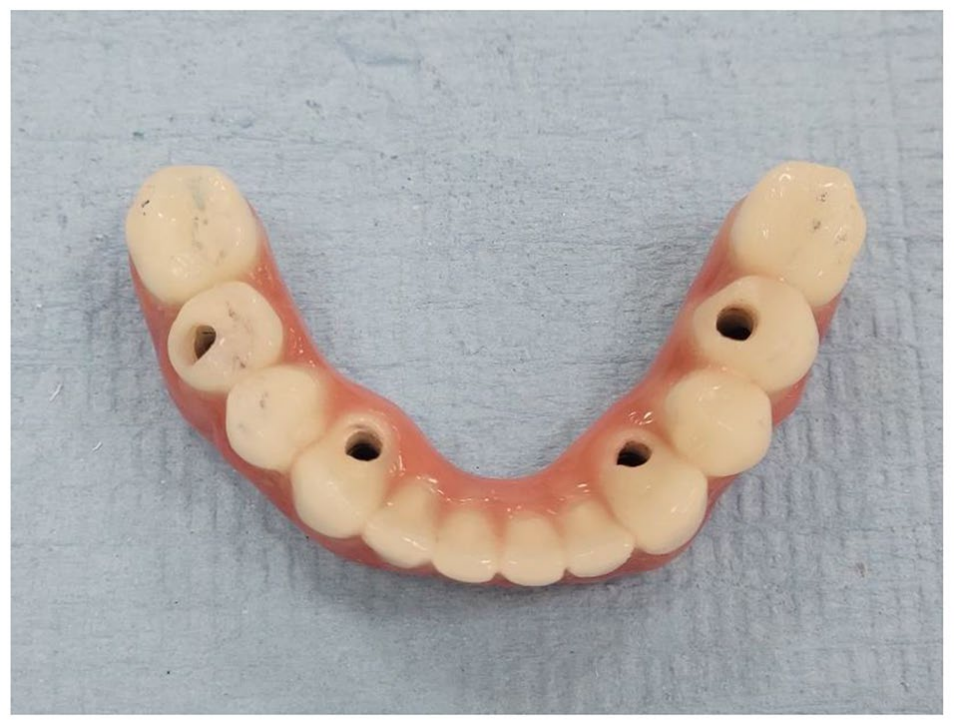
PEEK framework final prosthesis

**Fig. 3:**
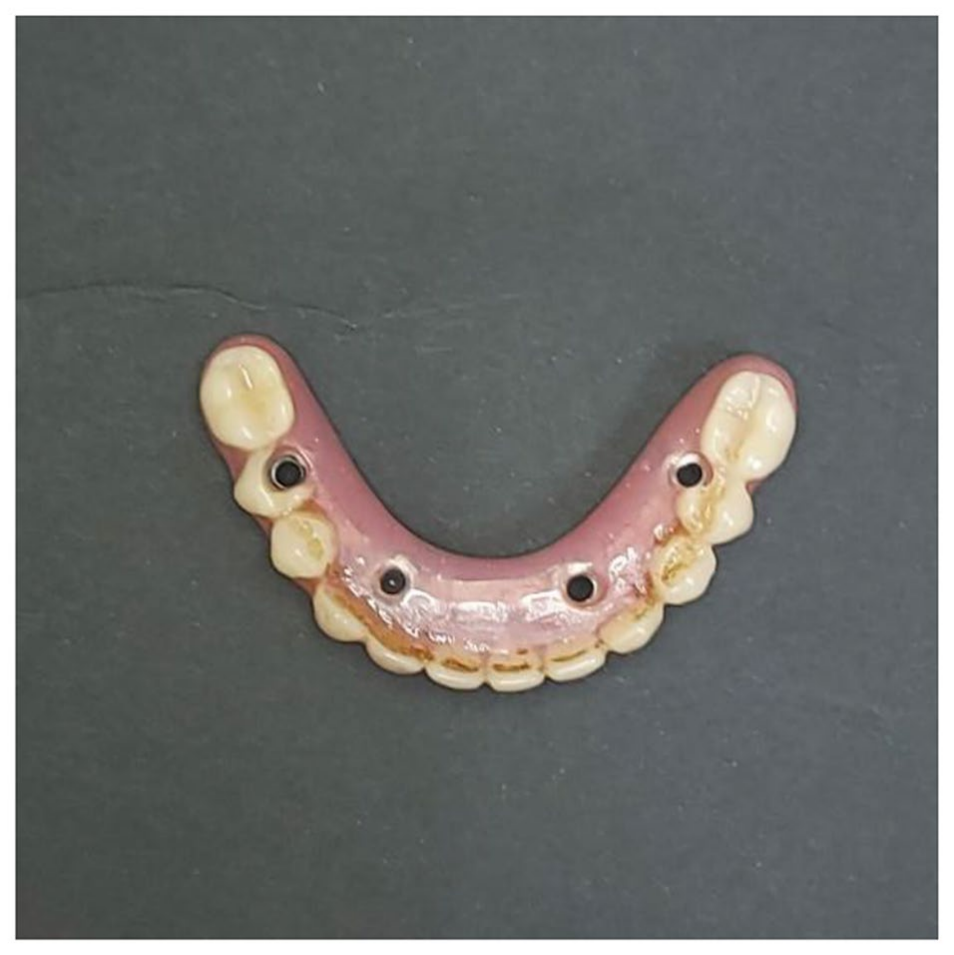
Metal framework final prosthesis

The frameworks were then cemented to the screw abutment cylinder using DTK cement (DTK-Klebr, Bredent GmbH & Co.). The passivity and fit of the frameworks were verified Intraorally using one screw test and periapical x-rays. For Group PK The teeth crowns were CAM milled following the CAD design of the resin try-in from high-impact polymethylmethacrylate (PMMA) material blocks (Novo.lign; Bredent GmbH & Co.). Then, an indirect light-polymerized nano-filled composite resin of a pink shade (Crea.lign; Bredent GmbH & Co.) was applied to estimate the gum tissue. (Fig. 2). The metal frameworks for Group PSM and Group SLM were then fastened to the definitive cast, and the teeth were placed. Wax contouring was done once the occlusion was improved. The goal of the final try-in was to assess aesthetics and jaw relationships. Heat-polymerized acrylic resin was used to process the mandibular prosthesis (Acrostone Dental Factory). (Fig. 3) After finishing and polishing, a laboratory remount was performed to adjust occlusion. The occlusion was adjusted to a lingualized occlusion scheme based on the patient’s centric relation [40]. The implant screw retained prosthesis was screwed to 18 Ncm following the manufacturer’s recommendations.

Using a CBCT and the approach outlined by Elsyad et al., the circumferential bone level on all implant faces was evaluated [41]. To achieve a high degree of measurement accuracy, the following scanning (iCAT next generation, Imaging sciences international (ISI), Hatfield, PA, USA) parameters were chosen: 120KvP, 5 mA, voxel size 0.25 mm, 14.7 s acquisition time, high-definition scan mode of 360° (total rotation), field of view (FOV diameter 16 cm, height 6 cm with a resolution of 0.157 × 0.157 mm. Each patient’s three-dimensional volumetric pictures that were captured and rebuilt were exported as DICOM files (Digital Imaging and Communications in Medicine) and examined with CyberMed’s OnDemand3DApp image analysis program [42].

**Fig. 4:**
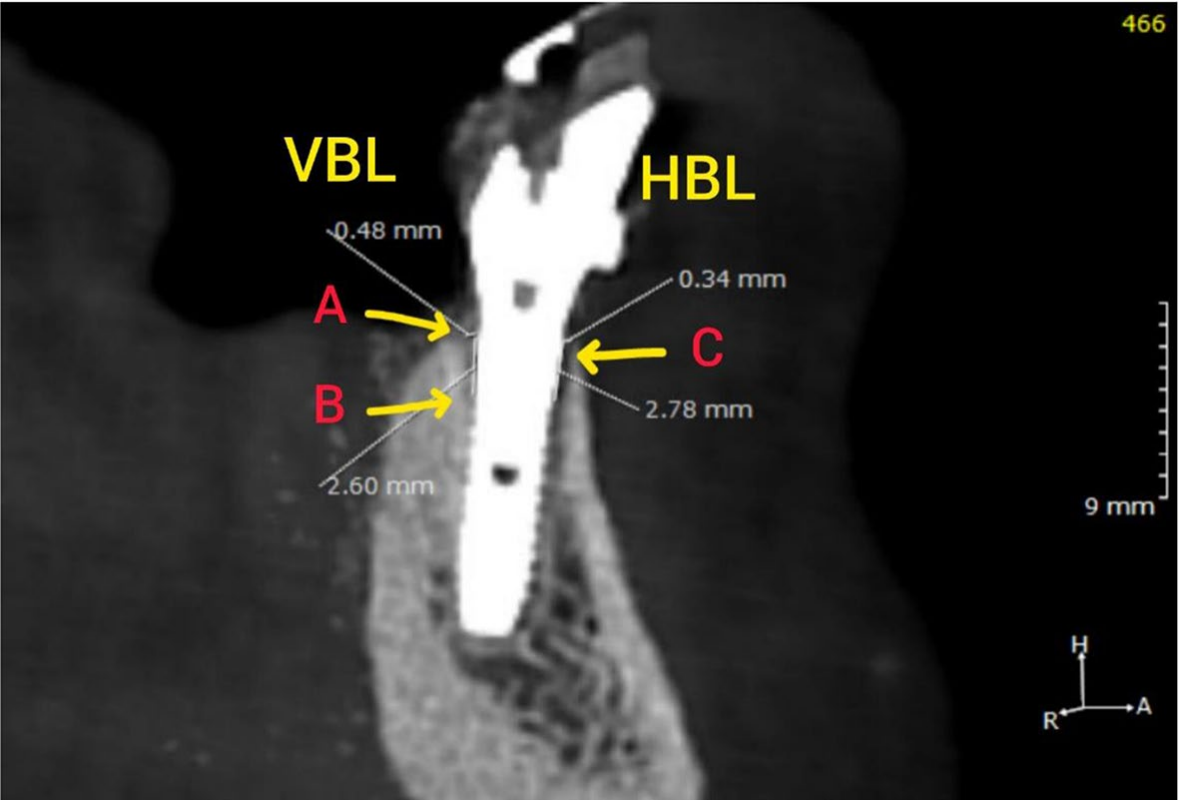
Measurements of circumferential peri-implant bone level on CBCT

By locating the center of the coronal portion of the implant, the three-dimensional position (X, Y, and Z) of the implant in the patient’s dental arch was determined. Then, using digital guidelines, horizontal planes (X and Y) at right angles to the long axes of each implant were reconfigured to produce two vertical transversal images as follows: a buccolingual image formed by the buccolingual implant image’s bisectional axis, and a mesiodistal image formed by the bisection of the alveolar crest and the implants mesiodistally. The four faces surrounding the implant were thus recognized in each transverse buccolingual image: buccal, lingual, distal, and mesial. The marginal bone levels, also known as the vertical and horizontal bone levels, were established on all four faces [43, 44]. By counting the millimeters between the component-implant junction (A) and the initial bone-implant contact (B), the vertical bone level was calculated. The perpendicular distance (in mm) between the implant and the marginal bone crest (point C) was used to calculate the horizontal bone level (Fig. 4).

The vertical and horizontal bone level subtractions at year 5 (T2), year 3 (T1), and year 1 (T0), respectively, correspond to the vertical and horizontal circumferential bone change parameters, where negative values signify bone loss between periods and positive values indicate bone gain or apposition [41, 42]. The mean bone level, which was calculated as the average of all implant faces, refers to the average horizontal and vertical bone level. The two anterior and two posterior implants’ mean measurements were calculated, and the mean was subsequently used for statistical analysis. Each scan’s image contrast and brightness were standardized by the software. To determine the reliability index of the measurements, a single-blinded assessor conducted the measurements in duplicate and in random order.

Data were analyzed using the SPSS program (SPSS v25.0; SPSS Inc). The test of normality was performed using Shapiro Wilk test. The data were normally distributed and presented as mean ± standard deviation for descriptive statistics. Two-way repeated measures analysis of variance (ANOVA) was used to compare vertical bone loss (VBL) and horizontal bone loss (HBL) between different groups, implant positions, and observation times followed by Tukey’s multiple comparisons if significant differences were detected. P was significant at 5%.

## Results

The results of vertical bone loss (VBL) using repeated measures ANOVA are presented in (Table 1). There was a significant difference in overall VBL between observation times (F (2,72) = 152.39, *P* < .001*), and group (F (2,72) = 247.642, *P* < .001*). However, there was no significant difference in overall VBL between positions (F (2,72) = 0.986, *P* = .324). The interaction groups*positions (F (2,72) = 106.447, *P* < .001*) and time*group (F (4,72) = 4.355, *P* = .003*) were significant. However, the interaction time*position and time*group*position were not significant.

**Table 1:** Summary of results of ANOVA for VBL

	Sum of squares	df	Mean square	F	*P* value
Time	0.099	2	0.050	152.399	< 0.001*
Group	0.161	2	0.081	247.642	< 0.001*
Position	0.000	1	0.000	0.986	0.324
Time * group	0.006	4	0.001	4.355	0.003*
Time * position	0.000	2	0.000	0.577	0.564
Group * position	0.069	2	0.035	106.447	< 0.001*
Time * group * position	0.003	4	0.001	2.092	0.091
Error	0.023	72	0.000		

*p is significant at 5% level of significance

Comparisons of VBL between groups and between evaluation times for the anterior and posterior implants are shown in (Table 2). For all groups, VBL increased significantly with time for both anterior and posterior implants. There was a significant difference in VBL between each 2-observation time. Also, for all observation times, there was a significant difference in VBL between groups for both anterior and posterior implants. For anterior implants, group PK showed the highest VBL, followed by Group SLM, and Group PSM showed the lowest VBL. For posterior implants, group PK showed the highest VBL, followed by group PSM, and group SLM recorded the lowest VBL. Multiple comparisons between each 2 groups are presented in the same tables. For all observations at anterior and posterior implants, there was a significant difference in VBL between every 2 groups except between group PSM and group SLM in all observations for posterior implants.

**Table 2:** Comparison of VBL between groups and observation times for anterior and posterior implants

	**T1**		**T2**		**T3**		***P*** value
	***X***	***SD***	***X***	***SD***	***X***	***SD***	
**Comparison of VBL between groups and observation times for anterior implants**							
Group PK	0.22 A, a	0.03	0.26 A, b	0.03	0.29 A, c	0.01	< 0.001*
Group PSM	0.06 B, a	0.02	0.09 B, b	0.01	0.12 B, c	0.02	< 0.001*
Group SLM	0.13 C, a	0.01	0.20 C, b	0.02	0.25 C, c	0.01	< 0.001*
P value	< 0.001*		< 0.001*		< 0.001*		
**Comparison of VBL between groups and observation times for posterior implants**							
Group PK	0.17 A, a	0.03	0.21 A, b	0.02	0.24 A, c	0.02	< 0.001*
Group PSM	0.13 B, a	0.01	0.16 B, b	0.01	0.21 B, c	0.02	< 0.001*
Group SLM	0.12 B, a	0.02	0.15 B, b	0.01	0.20 B, c	0.02	< 0.001*
P value	< 0.001*		< 0.001*		0.001*		

X; mean, SD; standard deviation. *p is significant at 5% level of significance. Different upper-case letters in the same column indicate significant differences between groups (Tukey, *p* < .05). Same upper-case letters in the same column indicate non-significant differences between groups (Tukey, *p* > .05). Different lower-case letters in the same raw indicate significant differences between observation times (Tukey, *p* < .05). Same lower-case letters in the same raw indicate non-significant differences between observation times (Tukey, *p* > .05)

Except for group SLM at T1, there was a significant difference in VBL between implant positions (Table 3). For groups PK and SLM, anterior implants recorded significantly higher VBL than posterior implants. For group PSM, posterior implants showed significantly higher VBL than anterior implants.

**Table 3:** Comparison of VBL between anterior and posterior implants

	T1		T2		T3	
	*X*	*S*D	*X*	*S*D	*X*	*SD*
	**Group PK**					
Anterior implants	0.22	0.03	0.26	0.03	0.29	0.01
Posterior implants	0.17	0.03	0.21	0.02	0.24	0.02
P value	< 0.001*		< 0.001*		< 0.001*	
	**Group PSM**					
Anterior implants	0.06	0.02	0.09	0.01	0.12	0.02
Posterior implants	0.13	0.01	0.17	0.01	0.20	0.02
P value	< 0.001*		< 0.001*		< 0.001*	
	**Group SLM**					
Anterior implants	0.13	0.01	0.20	0.02	0.25	0.01
Posterior implants	0.12	0.02	0.14	0.01	0.21	0.02
P value	0.165		< 0.001*		< 0.001*	

X; mean, SD; standard deviation. *p is significant at 5% level of significance

The results of horizontal bone loss (HBL) with repeated measures ANOVA are presented in (Table 4). There was a significant difference in overall HBL between observation times (F(2,72) = 148.126, *P* < .001*), groups (F(2,72) = 85.752, *P* < .001*), and positions (F(1,72) = 117.845, *P* < .001*). The interaction groups*positions (F(2,72) = 224.507, *P* < .001*). However, the interaction time*group, time*position, and time*group*position were not significant.

**Table 4:** Summary of results of ANOVA for HBL

	Sum of squares	df	Mean square	F	*P* value
Time	0.092	2	0.046	148.126	< 0.001*
Group	0.053	2	0.026	85.752	< 0.001*
Position	0.036	1	0.036	117.845	< 0.001*
Time * group	0.008	4	0.002	6.203	< 0.001*
Time * position	1.556	2	7.778	0.025	0.975
Group * position	0.139	2	0.069	224.507	< 0.001*
Time * group * position	0.001	4	0.000	1.164	0.334
Error	0.022	72	0.000		

*p is significant at 5% level of significance

Comparisons of HBL between observation times for anterior and posterior implants are presented in (Table 5). For all groups, HBL significantly increased after 5 years for both anterior and posterior implants. There was a significant difference in HBL between each 2 observations time.

**Table 5:** Comparison of HBL between groups and observation times for anterior and posterior implants

	**T1**		**T2**		**T3**		***P*** value
	***X***	***SD***	***X***	***SD***	***X***	***SD***	
**Comparison of HBL between groups and observation times for anterior implants**							
Group PK	0.22 A, a	0.02	0.24 A, b	0.02	0.28 A, c	0.01	< 0.001*
Group PSM	0.23 A, a	0.03	0.26 A, b	0.02	0.28 A, c	0.01	< 0.001*
Group SLM	0.15 B, a	0.03	0.20 B, b	0.01	0.27 A, c	0.02	< 0.001*
P value	< 0.001*		< 0.001*		0.214		
**Comparison of HBL between groups and observation times for posterior implants**							
Group PK	0.20 A, a	0.01	0.23 A, b	0.02	0.28 A, c	0.01	< 0.001*
Group PSM	0.08 B, a	0.01	0.11 B, b	0.01	0.14 B, c	0.01	< 0.001*
Group SLM	0.20 A, a	0.02	0.23 A, b	0.02	0.29 A, c	0.02	< 0.001*
P value	< 0.001*		< 0.001*		< 0.001*		

X; mean, SD; standard deviation. *p is significant at 5% level of significance. Different upper-case letters in the same column indicate significant differences between groups (Tukey, *p* < .05). Same upper-case letters in the same column indicate non-significant differences between groups (Tukey, *p* > .05). Different lower-case letters in the same raw indicate significant differences between observation times (Tukey, *p* < .05). Same lower-case letters in the same raw indicate non-significant differences between observation times (Tukey, *p* > .05)

Comparisons of HBL between groups for anterior and posterior implants are presented in (Table 5). For all observation times, there was a significant difference in HBL between groups for both anterior and posterior implants. For anterior implants, group PSM showed the highest HBL, followed by Group PK, and Group SLM showed the lowest HBL. There was a significant difference in HBL between every 2 groups except between group PK and group PSM. For posterior implants, group PK and group SLM showed the highest HBL, and group PSM showed the lowest HBL. There was a significant difference in HBL between every 2 groups except between group PK and group SLM.

Except for group PK at all observation times, there was a significant difference in HBL between implant positions (Table 6). For group PSM, anterior implants recorded significantly higher HBL than posterior implants. For group SLM, posterior implants showed significantly higher HBL than anterior implants.

**Table 6:** Comparison of HBL between anterior and posterior implants

	T1		T2		T3	
	***X***	***SD***	***X***	***SD***	***X***	***SD***
	**Group PK**					
Anterior implants	0.22	0.02	0.24	0.02	0.28	0.01
Posterior implants	0.20	0.01	0.23	0.02	0.28	0.01
P value	0.052		0.284		0.858	
	**Group PSM**					
Anterior implants	0.23	0.03	0.26	0.02	0.28	0.01
Posterior implants	0.08	0.01	0.11	0.01	0.14	0.01
P value	< 0.001*		< 0.001*		< 0.001*	
	**Group SLM**					
Anterior implants	0.15	0.03	0.20	0.01	0.27	0.02
Posterior implants	0.20	0.02	0.23	0.02	0.29	0.02
P value	< 0.001*		0.003*		0.014*	

X; mean, SD; standard deviation. *p is significant at 5% level of significance

## Discussion

This study was conducted since there was a lack of data in the literature available regarding the clinical performance of the Co-Cr framework produced using Pre-sintered soft metal block (PSM) and selective laser melting technology (SLM) on the peri-implant marginal bone or comparing it to other framework materials. According to Buzayan M. and Yunus N [45]. There is a connection between the stresses at the bone-implant interface and the alterations to the bone around the implant. Therefore, as the novel framework material that provides some resiliency in the prostheses framework may be able to lower stress concentration, it was investigated in this study whether or not they would affect bone loss. In this study, the peri-implant vertical and horizontal bone loss of PEEK and Co-Cr, frameworks for implant-supported prostheses made using (SLM) or milling from soft metal block (PSM), were examined. Additionally, the amount of bone lost from anterior and posterior implants was compared.

Several studies have reported using CBCT to measure the peri-implant bone changes around implants with an acceptable level of accuracy [41, 43, 44]. The disadvantages of CBCT, however, include a larger radiation dose when compared to traditional imaging methods and metal artifacts from beam hardening [46]. These artifacts, however, did not affect the measures of the peri-implant bone [44].

The null hypothesis of this retrospective study that no difference would be found between the three framework materials concerning the bone height change values around the dental implants of fabricated prostheses was rejected. The results of the study showed a significant increase in the VBL by time for both anterior and posterior implants for all groups, with the highest values observed in group PK (PEEK). This can be attributed to the low elastic modulus of the PEEK framework that was found to decrease the stress that occurred in the framework and increase that occurred in the trabecular bone region [8]. This may also be due to the material’s high degree of flexibility and the lack of a rigid framework. The prosthesis was made specifically using PMMA crowns, which exhibited less rigid biomechanical behavior. Furthermore, it was discovered that the trabecular bone region had a high-stress concentration of about 13 to 14 MPa as a consequence of the PEEK and PMMA combination [8]. These values are important to consider when choosing a framework material because it has been suggested that trabecular bone overload caused by a stress concentration value greater than 5 MPa may result in bone resorption [47].

Pre-sintered soft metal alloy (PSM) group showed higher values of posterior VBL than anterior VBL while the anterior VBL of (PSM) group recorded the lowest values compared to other groups. This may be attributed to the marginal accuracy and passive fit obtained from the milling of (PSM) blocks that consequently would minimize stresses on peri-implant bone [34, 48]. This finding coincides with Woo et al.’s study which found that the full-arch frameworks fabricated using soft-alloy milling exhibited a marginal accuracy that was comparable to those fabricated using hard-alloy milling [19]. These results also agree with several study findings that support the use of a rigid framework with multiple implants to achieve better stress distribution and reduce the stresses that may overload the peri-implant bone [49, 50].

Regarding peri-implant horizontal bone loss, it was found that peri-implant horizontal bone loss is primarily linked to an increase in pathological strain [51]. As a result, it has been suggested that there is a connection between preserving the soft tissue around implants and maintaining the horizontal bone level [52]. For both anterior and posterior implants in all groups, there was a significant increase in HBL throughout the evaluation period. This could be attributed to plaque accumulation, which was discovered to be a common problem among patients who had this kind of hybrid prosthesis [25]. According to Levartovsky et al. [53], there is a possibility of experiencing soft tissue recession and food impaction in full-arch screw-retained implant-supported prostheses, which aligns with the current observation.

Soft metal framework (Group PSM) showed the highest HBL anteriorly and this can be related to the marginal accuracy of the framework at this area with the smaller inter-implant distance anteriorly compared to the posterior implants that recorded the lowest HBL [54]. These results are in accordance with previous studies which proved that the fabrication protocol had a significant effect on the marginal discrepancy values [55, 56]. A study conducted by Daou and Baba [56] revealed that soft-milled Co-Cr has a lower marginal fit compared to the milled lost wax technique. Pasali et al. [57] suggested a possible reason that the higher misfit values in the soft block specimens is the milling procedure performed in the pre-sintered stage. During the sintering process, the contraction of the pre-sintered metal block is approximately 10%, producing a misfit if the amount of contraction is not calculated precisely [57]. In contrast to these results, Yang J and Li H [58] stated that soft CAD-CAM milling led to a more accurate marginal fit. The possible cause for this contrast is that their finding applied to single-unit metal copings, not a full arch framework.

Selective laser melting (Group SLM) showed the lowest anterior HBL. This can be attributed to the better marginal fit. In accordance with this result, Ortorp et al.‘s study [18], which compared the fit of Co-Cr restorations made using 4 different fabrication techniques (milled Co-Cr, milled wax with the lost wax method, conventional lost-wax method, and additional manufacturing direct laser metal sintering), revealed that SLM recorded the most accurate marginal fit. Although the surface of the hybrid prosthesis was roughened as a result of the additive manufacturing process employing SLM, which necessitated additional finishing steps, this was seen to be an additional benefit because it strengthened the mechanical bonding between the acrylic material and the framework [59].

The study’s limitations include the missing of a control group of patients with a conventional rigid Titanium framework. In addition to the use of a convenience sample, the difficulty quantifying bone remodeling due to artifacts caused by implants in the tomographic picture, and the limited amount of research using CBCT scans to examine bone tissue. Further prospective studies are needed to monitor the long-term bone changes around different framework materials and to be compared to the conventional rigid frameworks.

## Conclusion

Although using frameworks with some sort of resiliency for the full-arch implant-supported prosthesis; either by the material structure like PEEK or Co-Cr with novel manufacturing techniques (soft metal and selective laser melting); can alleviate stress concentration, they didn’t avoid the peri-implant bone loss. So, the application of these novel techniques subsidiary requires further periodic monitoring of the marginal bone level especially for the PEEK and SLM prosthesis.

Within the limitations of the study, Mandibular implant-supported fixed frameworks fabricated with either milling from PEEK or soft metal blocks, or addition manufacturing using laser melting technology exhibited significant vertical and horizontal bone height changes after 3 and 5 years.

Further long-term Comparative studies between rigid and flexible framework materials are still needed.

## Data Availability

The datasets used in the current study are available from the corresponding author upon request.
